# Antitumor Effects and the Potential Mechanism of 10-HDA against SU-DHL-2 Cells

**DOI:** 10.3390/ph17081088

**Published:** 2024-08-20

**Authors:** Yuanyuan Tian, Xiaoqing Liu, Jie Wang, Chuang Zhang, Wenchao Yang

**Affiliations:** 1 College of Bee Science and Biomedicine, Fujian Agriculture and Forestry University, Fuzhou 350002, China; tianyuan4601@126.com (Y.T.); lxq7597@163.com (X.L.); wangjie01092023@163.com (J.W.); chuangzhang12@163.com (C.Z.); 2 College of JunCao Science and Ecology (College of Carbon Neutrality), Fujian Agriculture and Forestry University, Fuzhou 350002, China

**Keywords:** survival, 10-HDA, SU-DHL-2 cell, label-free proteomics, complement and coagulation cascades pathway

## Abstract

10-hydroxy-2-decenoic acid (10-HDA), which is a unique bioactive fatty acid of royal jelly synthesized by nurse bees for larvae and adult queen bees, is recognized for its dual utility in medicinal and nutritional applications. Previous research has indicated that 10-HDA exerts antitumor effects on numerous tumor cell lines, including colon cancer cells, A549 human lung cancer cells, and human hepatoma cells. The present study extends this inquiry to lymphoma, specifically evaluating the impact of 10-HDA on the SU-DHL-2 cell line. Our findings revealed dose-dependent suppression of SU-DHL-2 cell survival, with an IC_50_ of 496.8 μg/mL at a density of 3 × 10^6^ cells/well after 24 h. For normal liver LO2 cells and human fibroblasts (HSFs), the IC_50_ values were approximately 1000 μg/mL and over 1000 μg/mL, respectively. The results of label-free proteomics revealed 147 upregulated and 347 downregulated differentially expressed proteins that were significantly enriched in the complement and coagulation cascades pathway (adjusted *p*-value = 0.012), including the differentially expressed proteins prothrombin, plasminogen, plasminogen, carboxypeptidase B2, fibrinogen beta chain, fibrinogen gamma chain, and coagulation factor V. The top three hub proteins, ribosomal protein L5, tumor protein p53, and ribosomal protein L24, were identified via protein–protein interaction (PPI) analysis. This result showed that the complement and coagulation cascade pathways might play a key role in the antitumor process of 10-HDA, suggesting a potential therapeutic avenue for lymphoma treatment. However, the specificity of the effect of 10-HDA on SU-DHL-2 cells warrants further investigation.

## 1. Introduction

Cancer remains a significant global health challenge, with an estimated 10 million fatalities in 2020, representing approximately one-sixth of all deaths, primarily due to prevalent malignancies such as breast, lung, colorectal, and prostate cancers [1]. Annually, diffuse large B-cell lymphoma (DLBCL) affects approximately 25,000 individuals worldwide, accounting for approximately 30% of non-Hodgkin lymphoma cases [2,3]. The etiology of DLBCL is multifactorial, with contributing factors ranging from genetic predispositions and viral infections (such as EBV) to environmental exposures such as agricultural pesticides and ionizing radiation, as well as physiological factors such as immunodeficiency and increased body mass index in young adults [4,5]. Over 60% of patients achieve remission through R-CHOP immunochemotherapy (including rituximab, cyclophosphamide, doxorubicin, vincristine, and prednisone), but other patients suffer from refractory or relapsed disease due to resistance to R-CHOP [4]. Consequently, extensive research efforts are dedicated to advancing DLBCL treatment modalities in clinical settings. The quest for safer therapeutics has propelled natural and dietary compounds to the forefront as promising substitutes for traditional chemotherapeutic agents. These endeavors include exploring the efficacy of chimeric antigen receptor (CAR) T cells, bispecific T-cell engagers, immunomodulatory drugs, checkpoint inhibitors, monoclonal antibodies, antibody–drug conjugates, targeted molecular pathway inhibitors, selective nuclear export inhibitors such as selinexor, and agents that modify epigenetic regulation [6,7].

Advances in the treatment of DLBCL have been marked by the exploration of novel therapeutic strategies and drug development. Recent studies have demonstrated that the combination of enzastaurin, a selective protein kinase C inhibitor, and ibrutinib, a Bruton’s tyrosine kinase inhibitor, synergistically impedes DLBCL cell survival and proliferation. This synergy manifests as reduced cell proliferation, enhanced apoptosis, G1 phase arrest, and diminished cell invasion, migration, and downstream signaling of ERK, mTOR, and PLCγ2 phosphorylation [8]. In addition, the novel synthetic compound BR101801, a dual inhibitor targeting DNA-PK and PI3Kδ combined with rafoxanide, has exhibited antitumor effects on DLBCL cells both in vitro and in vivo [9,10]. Various agents, including curcumin, 25-hydroxyvitamin D, disulfide, the survivin suppressant YM155, miR-145-5p overexpression, and quinacrine, have been shown to exert antitumor effects on DLBCL cells through distinct mechanisms. Specifically, curcumin impedes the proliferation of DLBCL subtypes by inducing G2 phase arrest and modulating the PPARγ and Akt/mTOR pathways [11]. According to meta-analytical data, elevated levels of 25-hydroxyvitamin D correlate with reduced cancer incidence and mortality [12]. Disulfide promotes apoptosis and cell cycle arrest at the G0/G1 phase in various DLBCL cell lines by inhibiting the NF-κB signaling pathway [13]. YM155 effectively inhibits the growth of several DLBCL cell lines by inducing apoptosis [14]. The upregulation of miR-145-5p has been shown to antagonize tumor growth by affecting the S1PR1/STAT3/AKT pathway [15]. Finally, quinacrine induces G0/G1 cell cycle arrest and apoptosis through the MSI2-NUMB signaling pathway in a dose-dependent manner [16]. These findings underscore the heterogeneous nature of DLBCL and the potential for targeted therapies to exploit distinct molecular vulnerabilities.

Bee products, including honey, propolis, bee pollen, bee venom, royal jelly, bee bread, beeswax, queen bee larvae, and drone pupa, are considered important natural products and functional foods due to their nutritional value and remarkable biological activities. Compared with Tualang honey (made by *Apis dorsata*), manuka honey has been shown to exert an inhibitory effect on the progression of N-methyl-N-nitrosourea-induced breast cancer [17]. This inhibition encompasses a reduction in the grade, size, angiogenesis, and vascular endothelial growth factor levels in MCF-7 and MDA-MB-231 breast cancer cell lines induced by 7,12-dimethylbenz(a)anthracene [18,19]. Additionally, the antioxidants present in Iranian natural honey counteract croton oil-mediated skin carcinogenesis [20]. A spectrum of cancer cell lines, including human hepatocellular carcinoma (HepG2) [21], colon cancer HT-29 [22], prostate cancer (PC-3) [23], bladder cancer (RT4, T24, 253J, and murine MBT-2) [24], pancreatic cancer (MIA PaCa-2 and BxPC-3) [25], lung cancer (NCI-H460) [26], melanoma (human A375, B16-F1, and B16-F murine) [27,28], renal cell carcinoma (786-O and 769-P) [29], and both acute and chronic leukemia (K562 and MV4-11) [30], have been reported to respond to the anticancer effects of honey. However, certain types of honey have been shown to have dual impacts on the viability of MCF-7 cells [31], potentially attributable to variations in the concentration of phenolic compounds [32]. These findings suggest a complex interaction between honey constituents and cancer cell dynamics, warranting further investigation to elucidate the mechanisms involved and optimize the therapeutic potential of honey in oncology.

**Figure 1:**
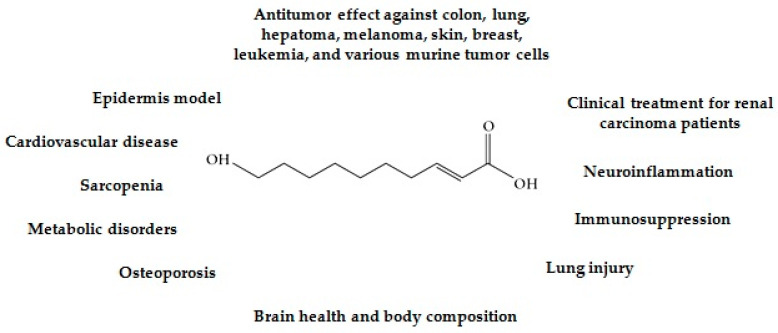
The structure and pharmacological properties of 10-hydroxy-2-decenoic acid (10-HDA).

Propolis, which is synthesized by *Apis mellifera* from various plant exudates and subsequently enriched with salivary enzymes, stands out for its pronounced anticancer properties. Research has consistently demonstrated the efficacy of propolis and its bioactive constituents in inhibiting the proliferation of a diverse array of human cancer cell lines, including those from oral, gastric, cervical, and colon cancers, as well as leukemia, skin, breast, prostate, lung, colorectal, urological, and lymphoma origins [33,34,35,36,37,38]. Bee pollen, composed of plant pollen collected by worker bees, also exhibits significant antitumor activity. It has been shown to combat a spectrum of cancers, including those of the prostate, breast, lung, stomach, liver, cervix, and ovary [39,40]. Bee venom, which is secreted by the venom glands of worker bees, is another potent inhibitor of cancerous growth and is effective against a variety of cell lines, such as those from breast, liver, melanoma, ovarian, lung, glioblastoma, gastric, hepatocellular carcinoma, leukemia, prostate, pancreatic, and non-small cell lung cancers [41]. Royal jelly is distinguished by its content of 10-HDA, a fatty acid implicated in its pharmacological activity (Figure 1) [42]. The antitumor potential of 10-HDA has been extensively documented across several cancer types, including colon, lung, hepatoma, melanoma, skin, breast, leukemia, and various murine models, as well as in clinical observations of renal carcinoma patients [42,43,44,45,46,47,48,49,50]. Nevertheless, the specific antitumor mechanisms and effects of 10-HDA on SU-DHL-2 cells remain to be elucidated.

To address this knowledge gap, we aimed to understand the link between the inhibitory effect of 10-HDA on SU-DHL-2 cells in vitro on cell survival, changes in protein expression determined using label-free proteomics, related gene expression trends, and conduction pathways.

## 2. Results

### 2.1. The Inhibitory Effects of 10-HDA on the Survival of SU-DHL-2 Cells

**Figure 2:**
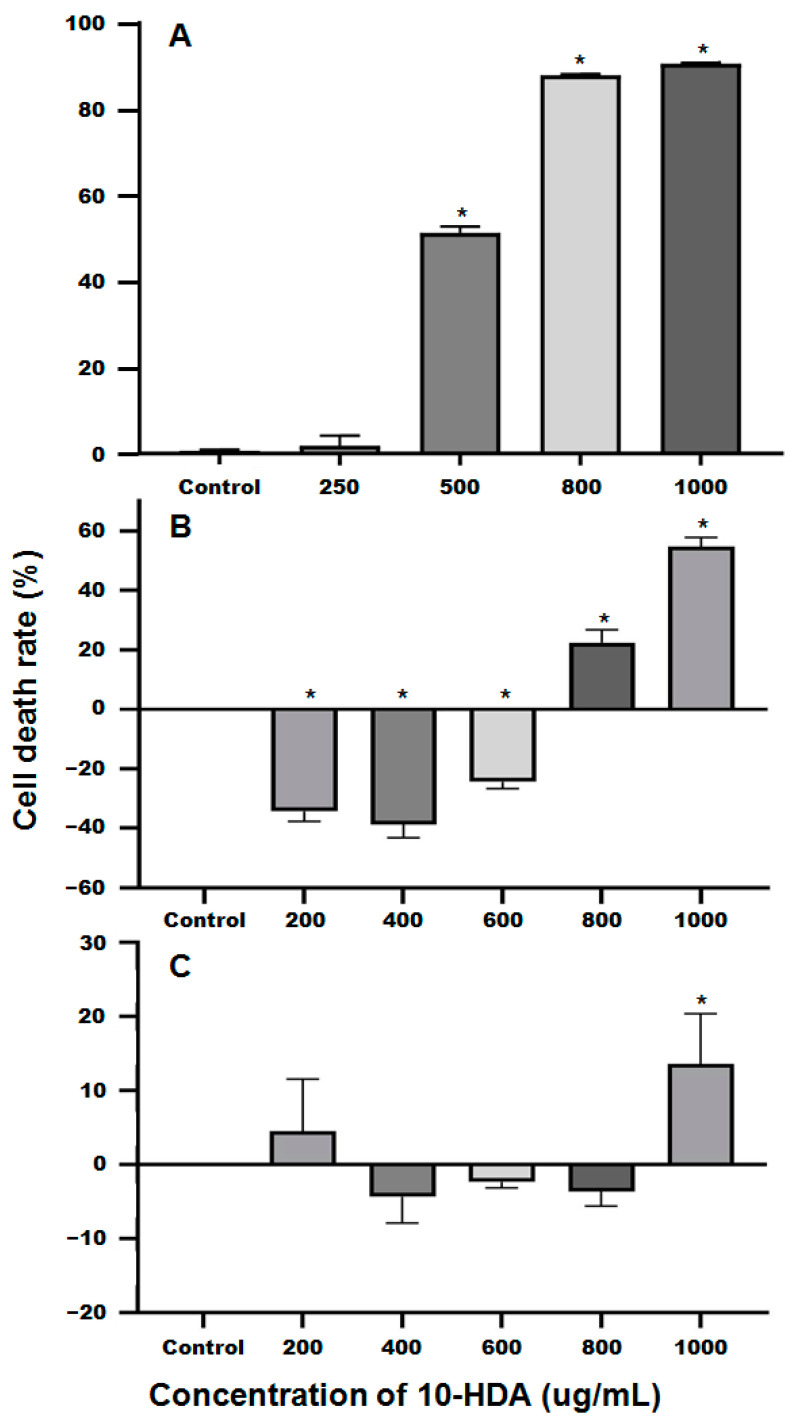
Cell death (%) of SU-DHL-2 cells (**A**), LO2 normal liver cells (**B**), and HSF normal human fibroblasts (**C**) treated with different concentrations of 10-HDA for 24 h. * indicates significant differences between the cell death rates of the treatment and control groups.

The control solvent (a complete culture medium with 0.02% ethanol, volume/volume) had no inhibitory effect on the survival of the SU-DHL-2 cells. However, significant inhibitory effects were observed in cells treated with 10-HDA at 250, 500, and 750 μg/mL (*p* < 0.01). Notably, there was no significant difference in cell death between the 750 μg/mL group and the 1000 μg/mL 10-HDA group. The data indicated that 10-HDA dose-dependently suppressed SU-DHL-2 cell survival (Figure 2A). The half-maximal inhibitory concentration (IC_50_) for SU-DHL-2 cells was determined to be 496.8 μg/mL after a 24 h incubation period.

At concentrations up to 600 μg/mL, 10-HDA did not exhibit cytotoxic effects but promoted survival in the standard liver cell line LO2, with an IC_50_ value of approximately 1000 μg/mL (Figure 2B). Similarly, no cytotoxicity was observed at concentrations up to 800 μg/mL in normal human fibroblast HSFs (Figure 2C, F_(5, 12)_ = 2.473, *p* = 0.0923).

### 2.2. Differentially Expressed Proteins in Treated Cells

**Figure 3:**
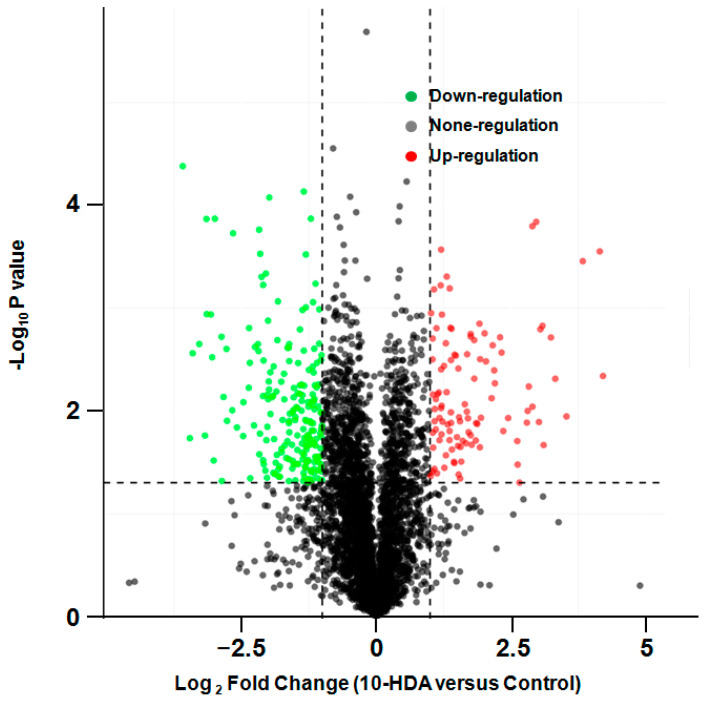
Volcano map of comparative protein expression determined using the label-free proteomic assay in SU-DHL-2 cells treated with 10-HDA (at the IC50 concentration) and untreated cells. Red dots represent significantly upregulated proteins, green dots represent significantly downregulated proteins, and black dots represent proteins whose expression did not significantly change.

The results of the label-free proteomic analysis of SU-DHL-2 cells treated with the solvent control and the IC_50_ of 10-HDA (496.8 μg/mL) are shown in Figure 3 as a volcano plot. This analysis revealed 147 proteins with significantly increased expression and 347 proteins with decreased expression. Additionally, 3405 proteins exhibited no significant change in their expression levels.

DEPs were mainly located in the nucleus (41.85%), cytoplasm (14.77%), mitochondria (11.38%), plasma membrane (6.46%), endoplasmic reticulum (5.54%), Golgi apparatus (4.92%), cytoskeleton (4.92%), lysosome (3.69%), extracellular space (3.08%), endosome (1.23%), centrosome (0.92%), peroxisome (0.62%), synapse (0.31%), and microsome (0.31%).

**Figure 4:**
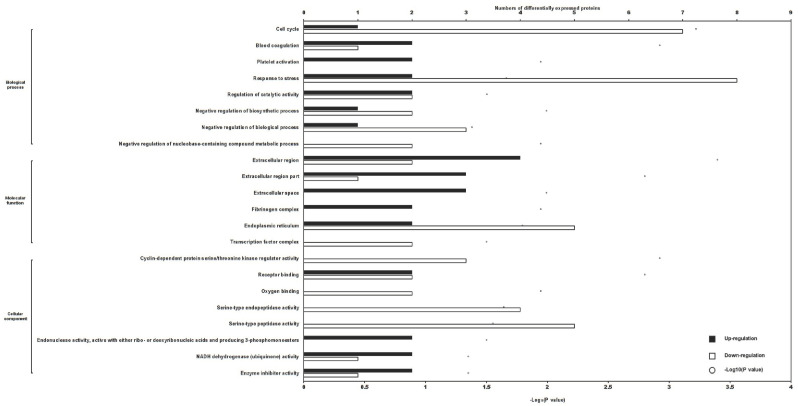
The GO enrichment terms of differentially expressed proteins (*p* < 0.05). The black and white bars indicate the number of upregulated or downregulated differentially expressed proteins in one term, respectively. The circles indicate the −log_10_ (*p*-value).

Gene Ontology (GO) enrichment analysis revealed that 93 DEPs were enriched in biological process, 28 DEPs in cellular component, and 113 DEPs in molecular function, of which 22 biological terms were significantly enriched (*p* < 0.05), as illustrated in Figure 4. The ‘response to stress’ category encompassed the greatest number of DEPs, with eight downregulated proteins and two upregulated proteins, followed by the ‘cell cycle’ category, which included seven downregulated DEPs and one upregulated DEP, and the ‘endoplasmic reticulum’ category, which included five downregulated DEPs and two upregulated DEPs.

Kyoto Encyclopedia of Genes and Genomes (KEGG) pathway enrichment analysis revealed a significant difference in the DEPs in a distinct pathway, namely, the complement and coagulation cascades pathway (adjusted *p* < 0.05, Table 1); other pathways showed no significant difference (adjusted *p* > 0.05).

**Figure 5:**
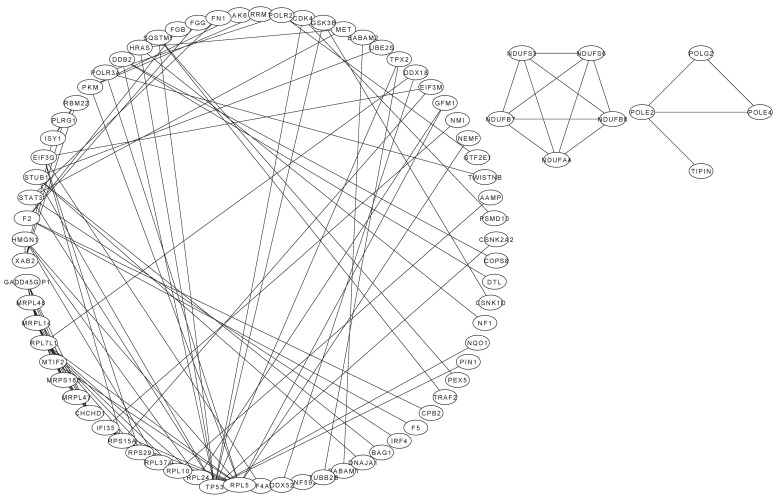
Protein–protein interactions of differentially expressed proteins (interaction score > 0.9). The characters indicate the gene names of differentially expressed proteins, and the lines between genes indicate that the differentially expressed proteins interact.

The PPI network of the DEPs with interaction scores exceeding 0.9 is depicted in Figure 5. The most prominent nodes within this network were represented by ribosomal protein L5, which interacts with 13 other proteins. This interaction was closely followed by the interaction of the tumor protein p53 with 12 proteins and the ribosomal protein L24 with 9 proteins.

### 2.3. Relative Expression of Selected Genes

**Figure 6:**
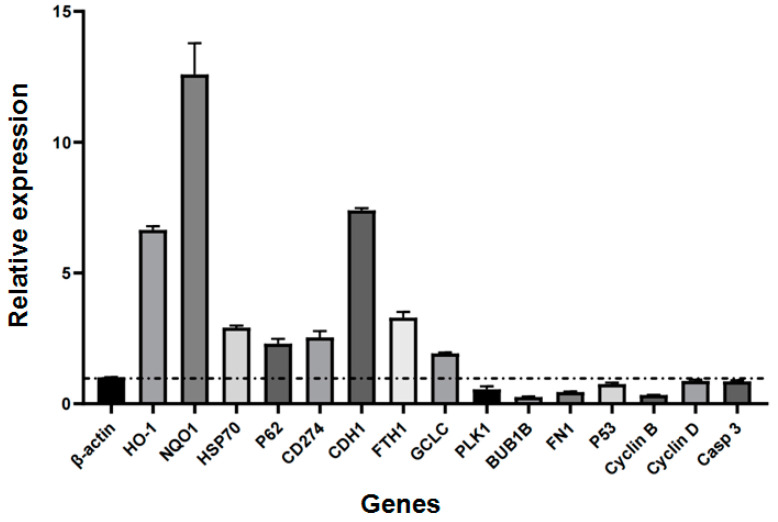
The relative expression of genes. Genes with a relative expression greater than 1 (dashed line) were upregulated, and those with a relative expression less than 1 were downregulated.

Figure 6 shows the relative expression levels of the selected genes. The genes *HO-1*, *NQO1*, *HSP70*, *P62*, *CD274*, *CDH1*, *FTH1*, and *GCLC* exhibited upregulated expression. In contrast, the genes *PLK1*, *BUB1B*, *FN1*, *P53*, *Cyclin B*, *Cyclin D*, and *Caspase 3* exhibited downregulated expression.

## 3. Discussion

The antitumor efficacy of 10-HDA has been documented across various cancer cell lines. The variability in IC_50_ values across different cancer cell lines and normal cell lines (Table 2) likely reflects the heterogeneity in cell density, determination methods, and differential sensitivity to 10-HDA [51,52]. A concentration of 200 μg/mL 10-HDA was found to be noncytotoxic to rat T cells from immunized animals (3 × 10^5^ cells/well for 4 days) according to a 3H-thymidine incorporation assay [51]. In the present study, the IC_50_ values for 10-HDA in normal liver cells (LO2) and HSF were approximately 1000 μg/mL and over 1000 μg/mL, respectively, as displayed in Figure 2B,C. Further experiments on the cytotoxicity of 10-HDA against other normal cell lines should be performed.

The survival inhibition mechanisms of 10-HDA against cancer cell lines appear to be multifaceted and influenced by the diversity of cancer types and the respective cell lines. Proteomic technology, a critical tool in contemporary research, has been extensively utilized to identify differentially expressed proteins in response to various treatments [37,54,55,56]. In SU-DHL-2 cells treated with 10-HDA, 494 DEPs were identified, 147 of which were upregulated and 347 of which were downregulated. KEGG pathway enrichment analysis revealed a significant association of these DEPs with the complement and coagulation cascades pathway (adjusted *p* < 0.05), and nonsignificant differences in the other pathways are depicted in Table 2.

This particular pathway is integral to the survival inhibition of SU-DHL-2 cells, notably influencing the transport mechanisms of the protein corona associated with magnetic PEI/siRNA complexes [57]. Furthermore, comprehensive serum proteomic analyses have underscored the importance of this pathway in the pathophysiology of meningioma, as evidenced by studies across various grades of the disease using multiple quantitative proteomic and immunoassay-based methodologies [58]. Similarly, quantitative proteomic analysis has highlighted the complement and coagulation cascades pathway as a significant factor in epithelial ovarian cancer [59], malignant ascites in hepatocellular carcinoma [60], and other malignancies, including hepatocellular carcinoma [60,61,62], bladder cancer [63], and ovarian cancer [64], as determined through bioinformatics analyses.

Additionally, this pathway has been identified as a potential biomarker for predicting the response to immunotherapy in patients with metastatic urothelial cancer [65], further demonstrating its relevance in cancer treatment strategies. The breadth of evidence across various cancer types underscores the ubiquitous role of this pathway in tumor biology and its potential as a therapeutic target.

DEPs associated with the complement and coagulation cascades pathway include vital factors such as prothrombin, various plasminogen isoforms, carboxypeptidase B2, fibrinogen beta and gamma chains, and coagulation factor V. Prothrombin, a critical plasma glycoprotein, comprises a gamma-carboxyglutamic acid (Gla) domain, two kringle domains, and a serine protease domain and is essential for blood coagulation. Recombinant human prothrombin kringles 1, 2, and 1-2 (rk-1, -2, -1-2) have been shown to inhibit tumor growth and metastasis in Lewis lung carcinoma [66]. Genetic ablation of the prothrombin gene in mice is associated with embryonic and neonatal mortality, underscoring its physiological importance [67,68]. Thrombin, generated from prothrombin through proteolytic cleavage, catalyzes thrombus formation and regulates the coagulation cascade [69]. It has been implicated in promoting tumor progression via fibrin formation and the activation of protease-activated receptors and platelets; hence, thrombin inhibitors have shown efficacy in treating malignancies such as 4T1 mammary adenocarcinoma in murine models [70] and ovarian cancer [71]. In the present study, thrombin activity was attenuated by the downregulation of prothrombin expression, which induced apoptosis in SU-DHL-2 cells.

Plasminogen, another DEP, is a serine protease precursor converted to active plasmin. Plasmin, along with matrix metalloproteinases activated by plasminogen, plays a pivotal role in the degradation of the extracellular matrix. This degradation process is crucial for tumor-related inflammation, leukocyte infiltration, cancer cell invasion, and metastasis [72], influencing angiogenesis and cell migration [73]. In this study, the downregulation of plasminogen resulted in reduced plasmin levels, contributing to the inhibition of SU-DHL-2 cell survival.

Carboxypeptidase B2, a critical enzyme within the complement and coagulation cascades pathway, plays a significant role in modulating inflammation by inactivating substances that act as activators and attractants for neutrophils. It has been identified as a prognostic indicator for oral squamous cell carcinoma [74] and a colorectal cancer biomarker [75]. Moreover, carboxypeptidase B2 has been recognized as a central protein in the plasma of breast cancer patients [76], and its suppression by siRNA has been shown to impede the invasion and migration of breast cancer cells, suggesting its potential as a therapeutic target [77]. In this study, the downregulation of carboxypeptidase B2 was associated with inhibited survival of SU-DHL-2 cells.

Fibrinogen, a complex glycoprotein, comprises alpha, beta, and gamma chains and plays a multifaceted role in tumorigenesis and cancer progression. The therapeutic application of exogenous fibrinogen has been demonstrated in the entrapment of OK-432 within the tumor stroma, leading to the regression of colorectal carcinoma [78]. Furthermore, fibrinogen-like protein 2 has been proposed as an immunotherapeutic target for brain tumors [79]. In breast cancer, both fibrinogen beta and gamma chains have been implicated as key factors in tumor progression and metastasis [80]. Downregulation of the fibrinogen gamma chain has been associated with reduced resistance to anthracycline chemotherapy in breast cancer [81], and silencing of this chain has been shown to markedly increase apoptosis and reduce the proliferation, invasion, and migration of cancer cells [82]. The influence of fibrinogen on SU-DHL-2 cells is complex, impacting multiple aspects of cell behavior and response to treatment.

Coagulation factor V is essential for regulating blood coagulation and exhibits both procoagulant and anticoagulant effects. Its role extends beyond hemostasis, as it has been identified as a novel marker for immune cell infiltration in breast cancer. It has potential as an immunological biomarker with therapeutic implications for the nexus of cancer, inflammation, and thrombosis [83]. Elevated levels of coagulation factor V in estrogen receptor-positive breast tumors have been correlated with improved relapse-free survival in patients [84]. Furthermore, coagulation factor V has demonstrated antitumor activity against MDA-MB-231 cells, potentially by inhibiting tissue factor-induced activation of protease-activated receptor 2 [85]. This protein has also been recognized as a prognostic biomarker for gastric and prostate cancers, potentially affecting the immune microenvironment and patient survival by modulating transforming growth factor (TGF)-beta signaling [86,87,88]. In this study, increased expression of coagulation factor V was associated with decreased survival of SU-DHL-2 cells, potentially through immunological mechanisms.

PPI analysis revealed that DEPs were enriched across various pathways, interacting synergistically with other proteins. Notably, ribosomal protein L5, tumor protein p53, and ribosomal protein L24 were the top three interacting proteins (Figure 5). Ribosomal protein L5, which serves as a chaperone for 5S rRNA, was shown to regulate the MDM2/MDMX–p53 cascade, thereby inhibiting tumor cell proliferation [89]. It also interacts with MDM2 or MDMX, which are pivotal for tumor suppression by modulating the activity of p53 [90]. The tumor suppressor p53, encoded by the most frequently mutated gene in human cancers, is central to the cellular response to diverse stressors, including DNA damage, hypoxia, nutrient deprivation, and oncogene activation [91]. As a ferroptosis regulator, p53 controls cell death processes, highlighting its dual function in modulating this form of cell death [92]. Additionally, p53 modulates the expression of ribosomal protein L24, a translation factor implicated in tumorigenesis, further illustrating the intricate network of interactions that govern cellular fate and cancer progression [93]. These proteins play important roles in inhibiting the survival of SU-DHL-2 cells.

In the treated cells, the expression of genes encoding the hop proteins *P53*, *PLK1*, *BUB1B*, *FN1*, *Cyclin B*, *Cyclin D*, and *Caspase 3* decreased. Notably, the expression levels of these genes did not correspond with the abundance of their respective proteins. These data showed that the PPIs of the DEPs and differentially expressed genes (DEGs) were inconsistent. The expression of genes was less strongly correlated with the survival inhibition of SU-DHL-2 cells. This contradiction between mRNA and protein expression levels was consistent with findings in our previous study and other studies in the literature [38,94]. The correlation between protein and mRNA expression levels is generally moderate, with a reported coefficient of approximately 0.5 [95]. This correlation tends to be weaker for mRNAs and their structurally stable protein counterparts [94], suggesting that posttranscriptional and posttranslational mechanisms may play significant roles in determining protein abundance.

The limitations of this study are as follows: The xenograft tumor model in nude mice did not succeed many times, which may be caused by fewer cancer stem cells in the SU-DHL-2 cell samples injected in nude mice. The limitations of this study include the use of metabonomics, transcriptomics, cancer stem cells, molecular docking, or other advanced methods to accurately evaluate the antitumor mechanism of 10-HDA against SU-DHL-2 cells for new drug development. The positive control group was excluded because of the different antitumor mechanisms of 10-HDA and antitumor drugs against SU-DHL-2 cells, such as 5-fluorouracil, which has a higher IC_50_ than 10-HDA against A549, NIC-H460, and NCI-H23 cells [47]. This missing positive control was also found in other reports [96,97,98]. More in-depth research on the cell cycle of SU-DHL-2 cells inhibited by 10-HDA can be performed in the future.

10-HDA inhibits the survival of SU-DHL-2 cells by regulating proteins involved in the complement and coagulation cascade pathways, including the differentially expressed proteins prothrombin, plasminogen, plasminogen, carboxypeptidase B2, fibrinogen beta chain, fibrinogen gamma chain, and coagulation factor V. The combination of 10-HDA or royal jelly and R-CHOP or other potential antitumor drugs is a potential treatment strategy for the SU-DHL-2 cell line.

The IC_50_s of 10-HDA against the normal liver cell line LO2 and normal human fibroblast line HSF were approximately 1000 µg/mL and greater than 1000 µg/mL, respectively. The lower specificity of 10-HDA against SU-DHL-2 cells should be considered. If we control the ADI of royal jelly according to the pharmacokinetics results or develop and apply a targeted sustained-release drug delivery system, then 10-HDA can be used for the practical treatment of cancer and enhance the proliferation of LO2 liver cells (B) and normal human fibroblasts (HSFs).

## 4. Materials and Methods

### 4.1. Determination of the IC50 of 10-HDA against SU-DHL-2 Cells

SU-DHL-2 cells (ATCC CRL-2956, purchased from Cellcook, Guangzhou, China) and LO2 normal liver cells were cultured in complete medium (composed of 89% modified RPMI-1640 basal medium (Wuhan Pricella Biotechnology Co., Ltd., Wuhan, China), 10% fetal bovine serum (Cellmax Bio Co., Ltd. Lanzhou, China), and a 1% penicillin and streptomycin mixture (HyClone Biochemical Products Co., Ltd. Shanghai, China)) in a 5% CO_2_ humidified incubator at 37 °C (C150, Binder, Tuttlingen, German). Normal human HSF fibroblasts were cultured in complete medium (composed of 89% high-sugar DMEM purchased from Procell Life Technology Co., Ltd., Wuhan, China; 10% FBS; and a 1% penicillin and streptomycin mixture). The other chemicals and incubators used were the same as those described in our previous report [38].

Next, 50 milligrams of 10-HDA was dissolved in 100 µL of anhydrous ethanol. The 10-HDA solution was gradient diluted to 250, 500, 750, and 1000 µg/mL (the final concentration of 10-HDA in the complete medium) using complete culture medium until ready for use.

The concentration of SU-DHL-2 cells was adjusted to 3 × 10^6^ cells/well in 96-well plates. Different concentrations of 10-HDA solution and a complete medium containing 0.02% ethanol (*v*:*v*, solvent control, equal to ethanol in 1000 µg/mL 10-HDA solution) were added after 24 h of incubation. After 24 h of further incubation, the cell suspensions were collected to determine cell viability with a CCK8 kit (purchased from Dojindo, Kumamoto, Japan) at 450 nm using a microplate reader (1510, Thermo Fisher, Waltham, MA, USA). The percentage of SU-DHL-2 cells that died due to 10-HDA treatment was calculated as the OD_450_ of the control group minus the OD_450_ of the treated group and then divided by the OD_450_ of the control group, which was multiplied by 100%. The IC_50_ of 10-HDA against SU-DHL-2 cells for 24 h was calculated using GraphPad Prism 8.4.3 for Windows (GraphPad Software, Inc., La Jolla, CA, USA). Similarly, LO2 normal liver cells and HSF normal human fibroblasts were treated with 10-HDA solution at 200, 400, 600, 800, and 1000 µg/mL (the final concentration of 10-HDA in complete medium) for determination of the IC_50_ values.

### 4.2. Proteomic Determination of Differentially Expressed Proteins in Cells Following Different Treatments

The SU-DHL-2 cells (3 × 10^6^ cells/well in 96-well plates) were cultured with 10-HDA at the IC50 (496.8 μg/mL) or the solvent control (0.02% ethanol in complete medium) in a 5% CO_2_ humidified incubator at 37 °C for 24 h of incubation. The cells were collected and subjected to the same procedures as those described in our previous report [38]. The concentration of the total proteins extracted from cells frozen in liquid nitrogen was determined using Coomassie brilliant blue staining (CBB-G250). Then, the spectra of proteins in SU-DHL-2 cells were determined by Novgene Biotech Co., Ltd., Beijing, China.

### 4.3. Detection of the Relative Expression of Genes

The relative expression of genes encoding hub proteins (top 10 proteins) involved in protein–protein interactions (PPIs), namely, *HO-1*, *NQO1*, *Hsp70*, *p62*, *CD274*, *CDH1, PLK1*, *BUB1B*, *p53*, *Cyclin B*, *Casp 3*, and *Cyclin D*, was determined via RT–PCR using a C1000 Touch Thermal Cycler (Bio-Rad). The RT–PCR procedure was performed as previously described [38]. Primers (Table 3) were designed via the NCBI free online primer design platform with β-actin as the internal reference gene.

### 4.4. Statistical Analysis

All experiments were performed in triplicate, and the results are expressed as the mean ± standard error. The cell death (%) was transformed to arcsin (degree) values (according to the formula: arc sin√*p*) before ANOVA, which was performed using GraphPad Prism 8.4.3 for Windows (GraphPad Software, Inc., San Diego, CA, USA) to analyze the significance of differences (*p* < 0.01: highly statistically significant differences between the different treatment groups and *p* < 0.05: statistically significant differences).

All of the data obtained for differentially expressed proteins (DEPs) via a label-free technique were analyzed. The spectra obtained from LC–MS/MS were searched against the UniProt database using Proteome Discoverer 2.2 (Thermo) with credibility of more than 99% peptide spectrum matches. Proteins containing 1 or more unique peptides and with a probability of false discovery less than or equal to 1% were identified. The protein quantitation results were statistically analyzed with t-tests using GraphPad Prism 8.0.2 for Windows. DEPs were proteins with significantly different quantities between the treated group and the control group (*p* ≤ 0.05 and fold change (FC) ≥ 2 or FC ≤ 0.5). All DEPs were sent to the Gene Ontology database (GO, http://www.geneontology.org/ (accessed on 5 January 2024)) to calculate the number of proteins in each term. A hypergeometric test was applied to find GO terms that were the most significantly enriched in DEPs compared with all protein backgrounds. Kyoto Encyclopedia of Genes and Genomes (KEGG) was used to analyze the enriched pathways (http://www.genome.ad.jp/kegg/ (accessed on 5 January 2024)). The PPIs of the DEPs were determined using the String-db server (http://string.embl.de/ (accessed on 5 January 2024)), in which the minimum required interaction score was >0.9. Then, the data exported from STRING-db were loaded into Cytoscape software (version 3.9.1; JAVA: 11.0.6 by AdoptOpenJDK) to construct the PPI network diagram.

The relative gene expression values are represented by the ratio of the expression of a gene in 10-HDA-treated cells to that in the solvent control group cells.

## Figures and Tables

**Table 1:** The DEPs were enriched in significantly different pathways (*p* < 0.05).

Pathway	*p*-Value	Adjusted *p*-Value	Differentially Expressed Proteins	
			Upregulated	Downregulated
Complement and coagulation cascades	4.9 × 10^−5^	0.0124	A0A0A0MRJ7, P02675, C9JC84	P00734, A0A0F7G8J1, B2R7F8, Q96IY4
Basal cell carcinoma	0.0013	0.1615	A0A3B3ITW1, Q92466, Q6TKP8	A0A0U1RQC9
Mineral absorption	0.0028	0.2324	B2R7U4, P02795, P04733, A0A140VJP7	A0A0U1RQC9
Neuroactive ligand–receptor interaction	0.0037	0.2360	-	P00734, A0A0F7G8J1, B2R7F8
Pathways in cancer	0.0131	0.5780	K7EP08, P15559, A0A3B3ITW1, B2R7U4, P02751, A0A024R728, Q92466, P63218, Q6TKP8, A0A2 × 0SFF5	B3KT21, A0A0U1RQC9, Q9HAV0, U6FVB0, X5D945, P11802, A8K725, P33552, A0A024R8H5, Q5T178, A0A024QYW7, B3KNJ3
Small-cell lung cancer	0.0147	0.5780	P02751, Q92466	A0A0U1RQC9, P11802, P33552, A0A024R8H5, Q5T178
*Staphylococcus aureus* infection	0.0161	0.5780	C9JC84	A0A0F7G8J1, B2R7F8
Pyrimidine metabolism	0.0210	0.6627	P36954	P23921, D6W4Z6, Q9BZX2, A0A024R8N6, O14802, A0A5F9ZHU7, P56282, Q3B726, Q9NR33, Q7Z3R8, A8K9A5
Melanoma	0.0285	0.7929	A0A024R728, Q92466	A0A0U1RQC9, X5D945, P11802
Thyroid cancer	0.0346	0.7929	Q92466	A0A0U1RQC9, U6FVB0, X5D945
Bladder cancer	0.0346	0.7929	-	A0A0U1RQC9, X5D945, P11802, B3KNJ3

**Table 2:** The IC_50_s values of 10-HDA in different cell lines.

Cell Type	Cell Line	IC_50_ Value (μg/mL)	Method	References
Lung cancer cell	A549	4.22	CCK-8	[43]
Lung cancer cell	NCI-H460	8.20	CCK-8	[43]
Lung cancer cell	NCI-H23	8.34	CCK-8	[43]
Human hepatoma cell	HepG2	59.6	MTT	[44]
Human colorectal adenocarcinoma cell	CaCo-2	37.5	MTT	[50]
Breast cancer cell	MDA-MB231	651.88	-	[53]
Breast cancer cell	MDA-MB436	949.88	-	[53]
Breast cancer cell	HCC1937	979.68	-	[53]
Breast cancer cell	MCF-7	972.225	-	[53]
Human epithelial breast cell	MCF-10a	931.25	-	[53]
Human normal liver cell line	THLE-3	106.4	MTT	[44]
African green monkey kidney cell	Vero	3445.63	-	[53]
Fibroblast	NIH3T3 (Wt)	1862.5	-	[53]
Fibroblast	BRAF V600E mutation	1303.75	-	[53]
SIRC (Statens Seruminstitut Rabbit Cornea) cell	SIRC	2.38	MTT	[52]

**Table 3:** Primers utilized for the RT–PCR system.

Primer	Sequences (5′→3′)
*β-actin-F*	GATCATTGCTCCTCCTGAGC
*β-actin-R*	ACTCCTGCTTGCTGATCCAC
*HO-1-F*	TCTTGGCTGGCTTCCTTACC
*HO-1-R*	GGATGTGCTTTTCGTTGGGG
*NQO1-F*	TGAAAGGCTGGTTTGAGCGA
*NQO1-R*	TCCAGGCGTTTCTTCCATCC
*GCL-F*	AGGTCAAACCCAACCCAGT
*GCL-R*	TGTTAAGGTACTGAAGCGAGG
*BUB1B-F*	GGATGGGTCCTTCTGGAAACT
*BUB1B-R*	GTGGCCTCATCATTGGCATTC
*FTH1-F*	CAGAACTACCACCAGGACTCA
*FTH1-R*	TCAAAGCCACATCATCGCGG
*HSP70-F*	GTGTAACCCCATCATCAGCG
*HSP70-R*	GCTCCAAAACAAAAACAGCAATCT
*p62-F*	TACCAGGACAGCGAGAGGAAG
*p62-R*	ATCCTTTCTCAAGCCCCATGT
*Cyclin B-F*	GATACTGCCTCTCCAAGCC
*Cyclin B-R*	GCACACAATTATTCTCAAGTTGTC
*Cyclin D-F*	GCCGGGGACCGAAACT
*Cyclin D-R*	GCAGTGGCGAAGTGTTTACAAAG
*CD274-F*	TTTGCTGAACGCCCCATACA
*CD274-R*	TCCAGATGACTTCGGCCTTG
*CDH1-F*	GCTGGACCGAGAGAGTTTCC
*CDH1-R*	CAAAATCCAAGCCCGTGGTG
*p53-F*	ACACGCTTCCCTGGATTGG
*p53-R*	TCATCCATTGCTTGGGACGG
*Casp 3-F*	CTCTGGTTTTCGGTGGGTGT
*Casp 3-R*	CTTCCATGTATGATCTTTGGTTCC
*FN1-F*	CAAGCATGTCTCTCTGCCAAG
*FN-R*	CAGAACAGGCAATGTGCAGC
*PLK1-F*	CCTGCACCGAAACCGAGTTA
*PLK1-R*	ACCTCGAAACTGTGCCCTTT

## Data Availability

Data are contained within this article.
